# Smart monitoring technology to support home-based dementia care: Market-specific business model development and implementation considerations in the Netherlands

**DOI:** 10.1177/20552076251331825

**Published:** 2025-03-28

**Authors:** Christian Wrede, Annemarie Braakman-Jansen, Lisette van Gemert-Pijnen

**Affiliations:** 1 Centre for eHealth and Wellbeing Research, Department of Psychology, Health & Technology, 3230 University of Twente, Enschede, Netherlands

**Keywords:** Remote monitoring, assistive technology, dementia, ageing in place, implementation, business modelling

## Abstract

**Background:**

Remote monitoring (RM) technology can support home-based dementia care by enabling (in)formal caregivers to monitor the health and safety of people with dementia remotely. However, sustainable implementation is challenged by a lack of viable business models and a limited understanding of the markets in which these models might operate. This study aimed to (1) develop business models for RM technology in the Dutch consumer-, healthcare-, and social support market, and (2) identify entrepreneurial opportunities and challenges of implementing RM technology via those markets.

**Methods:**

Semistructured interviews and focus groups were conducted among three stakeholder groups (*n* = 20) including care providers (*n* = 8), financiers of care and support (*n* = 6), and technology providers (*n* = 6). The sessions addressed the Business Model Canvas and entrepreneurial opportunities and challenges of implementation for each market. All sessions were analyzed thematically.

**Results:**

The analysis resulted in three distinct business models which specify how RM technology for home-based dementia care could be sustainably implemented in the a) consumer market, b) healthcare market, and c) social support market. Possible entrepreneurial opportunities and challenges of implementing RM technology were identified per market. Topics dealt with, for instance, reaching potential user groups, time to market, the complexity of structural reimbursement, scalability, and alignment with future care trends.

**Conclusions:**

Our findings add to the limited research on the entrepreneurial side of implementing dementia care technology. The proposed business models and insights into market-specific considerations can guide developers in refining implementation strategies and selecting suitable markets for RM technology and other home care technologies.

## Introduction

### Background

Due to population ageing, the global prevalence of dementia is expected to almost triple by 2050.^1^ Currently, every three seconds someone in the world is diagnosed with dementia.^1^ Most people with dementia (PwD) prefer to live at home for as long as possible, despite the challenges posed by their condition.^2^ In response to this preference, along with a growing shortage in nursing home capacity,^3^ care policies have shifted toward a greater reliance on home-based care.^2,4^ Informal caregivers such as spouses, children, or other family members will likely have to provide more care and support—often from a distance—while the number of informal caregivers per PwD is expected to decrease.^5^ In the Netherlands, this has resulted in 56% of informal caregivers of PwD who already feel heavily burdened.^6^ Due to a shrinking workforce, formal caregivers involved in the home care of PwD face an increased workload too,^7,8^ which requires them to use their time more effectively.

To support formal and informal home-based dementia care, there is a need for innovative solutions, including those from the field of assistive technology.^9,10^ The use of remote monitoring (RM) technology enables (in)formal caregivers to remotely monitor the health and safety of PwD. By detecting critical events such as falls, nighttime issues, or deviations in self-care, RM technology is expected to provide caregivers with a sense of security and facilitate timely interventions, which could help to delay the institutionalization of PwD.^11–15^ Technically, RM technologies are advancing and becoming increasingly unobtrusive in several ways: First, there is a shift from sensors that require attachment to the body (wearables) or household equipment (e.g., sensors on doors or fridges) to contactless sensors (e.g., smart energy meters, motion sensors utilizing infrared, radar, or WiFi-CSI).^16,17^ Second, there is a trend to move away from active sensors (which require user interaction) to passive sensors (which operate without user interaction).^18^ Finally, the integration of AI is enhancing the intelligence of RM systems, enabling them not only to detect incidents like falls but also to predict them, thus opening up new opportunities for prevention.^19,20^

Despite its potential, RM technology for home-based dementia care can only realize its benefits if it can be sustainably implemented in practice which has, however, been found to be complex.^21,22^ In our previous research, we investigated how home care organizations can achieve successful implementation of RM technology for PwD^11^ and explored factors that are associated with user acceptance.^23^ Until now, much less attention has been devoted to the entrepreneurial side of implementation. In particular, how developers can build viable business models that can successfully bring novel RM technology into practice. A business model can be defined as “the rationale of how an organization creates, delivers, and captures value.”^24^ It describes how an eHealth service is introduced to the market and how it is envisioned to sustain, both organizationally and financially, without short-lived subsidies.^25,26^ Generally, published academic or industry research on business models for dementia care technology is rare and has so far mainly focused on online interventions for informal caregivers.^27,28^

Business models do not operate in a vacuum but are typically tailored to a specific implementation market.^24^ In the Netherlands, three main markets for assistive technologies exist:^29^ (1) the consumer market (where the technology becomes a consumer product), (2) the healthcare market (where the technology becomes part of professional care delivery), and (3) the social support market (where the technology becomes part of municipal support services). In the context of RM technology for home-based dementia care, published examples of potentially viable business models for these three markets are almost nonexistent—particularly those developed with input from relevant stakeholders. Such examples could help developers in designing or refining their own business model or implementation strategy. Furthermore, there is limited insight into the entrepreneurial opportunities and challenges of implementing RM technology via the consumer-, healthcare-, and social support market. It is likely that each market brings specific points of attention for developers, as they differ in terms of target group, potential payers, and complexity.^29^ Expanding this knowledge could help developers in selecting the most suitable implementation scenario for RM technology, based on relevant stakeholder perspectives.

### Aim of the study

The aim of this study was to develop viable business models for RM technology in home-based dementia care and to better understand the specific markets where these models could operate. By involving relevant stakeholders from aged care, business, and care financing, we aimed to:

1. Develop business models for the sustainable implementation of RM technology via three Dutch markets: (a) the consumer market, (b) the healthcare market, and (c) the social support market.
2. Outline possible entrepreneurial opportunities and challenges of implementing RM technology via the consumer-, healthcare-, and social support market.

## Method

### Study design

A qualitative research design was applied in this study. Data were collected using semistructured interviews and focus groups with relevant stakeholders (see Appendix 1 for the completed COREQ checklist).

### Study setting

**Figure 1.**
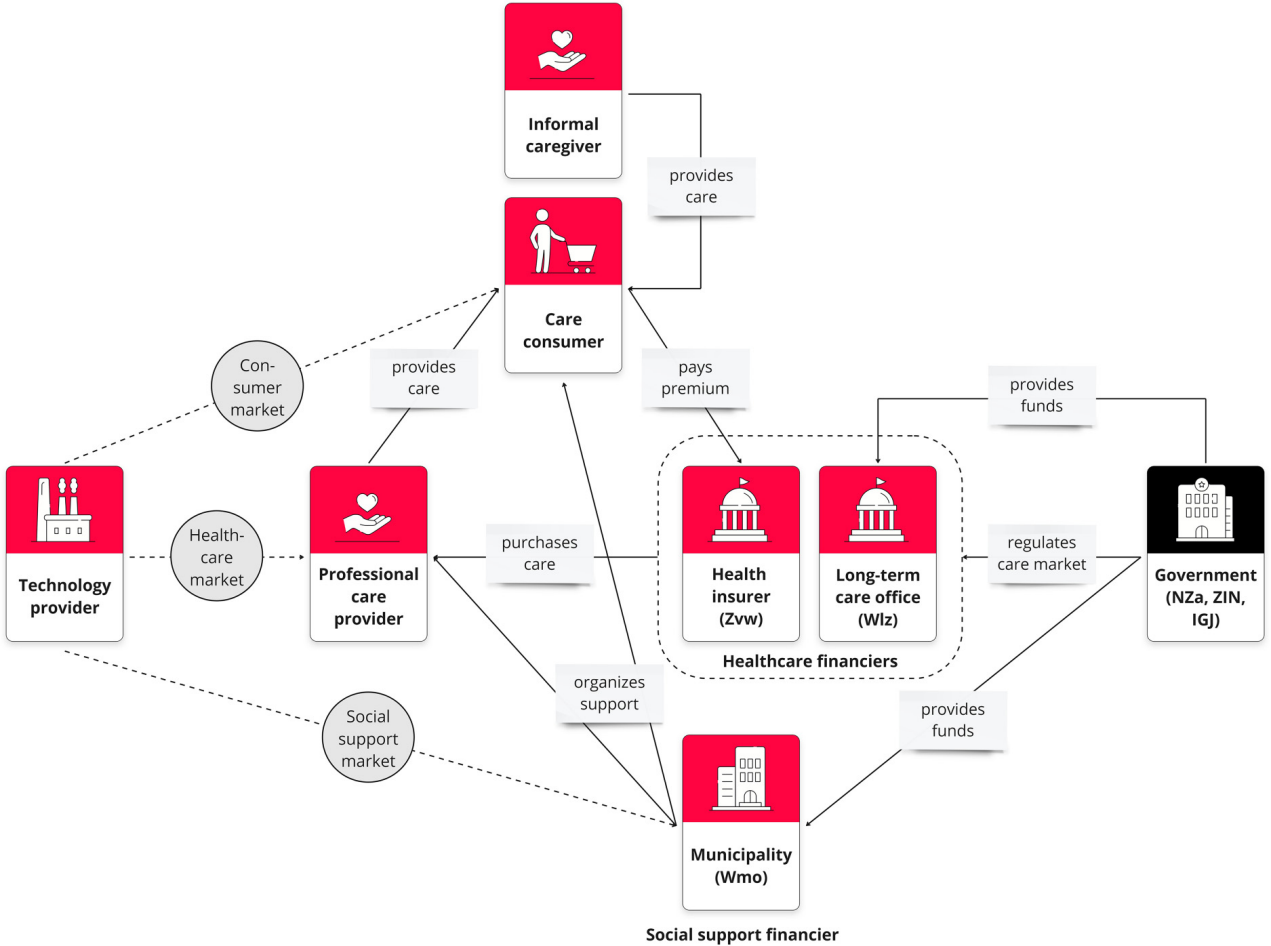
Simplified overview of main parties in the Dutch care system and different scenarios for implementing RM technology.

*Notes:* Zvw = Health Insurance Act; Wlz = Long-term Care Act; Wmo = Social Support Act; NZa = Dutch Healthcare Authority; ZIN = National Healthcare Institute; IGJ = Dutch Healthcare Inspectorate; RM = remote monitoring.

The study was conducted within the context of the Dutch care system. Figure 1 depicts a simplified general overview of the main parties involved and their interrelations. The core of the Dutch care system is formed by the “triangle” between (a) professional care providers, (b) care consumers (who pay premiums) and their informal caregivers, and (c) the care financiers (who purchase care from professional care providers).^29,30^ The two main care financiers include 1) private, competitive health insurers who are obliged to accept all citizens and execute the Health Insurance Act (Zvw) covering, for example, primary care, hospital care, or district nursing, and 2) government-funded long-term care offices which execute the Long-term Care Act (Wlz) covering, for example, residential care or intensive home care.^30,31^ The Dutch government regulates the care market and determines which care is reimbursed. Health insurers and long-term care offices have freedom to determine in which form that care is provided, searching for the best (evidence-based) care for the lowest price.^29,30^ Municipalities, on the other side, receive governmental funds to organize local support under the Social Support Act (Wmo). This also includes solutions to help older adults live at home for longer and to support their informal caregivers (e.g., day care, household help, or assistive devices).^29,30^ Support can be provided directly by municipalities, via care and welfare organizations, or through a personal budget allocated to citizens.^29,30^

### Participant recruitment

For this study, we invited three stakeholder groups that play a key role in implementing RM technology in home-based dementia care: care provider representatives, potential payers (health insurers, long-term care offices, municipalities), and technology providers (see Table 1 for a specification of included participants). We based the inclusion of participants on outcomes of a stakeholder identification survey we conducted previously within our overarching project on RM technology for home-based dementia care.^11^ Participants were invited via email using the research team's professional network and snowball sampling. Subsequently, appointments were scheduled for interview- and focus group sessions. All participants provided informed consent before the study.

**Table 1.** Overview of included participants (*n* = 20).

Stakeholder group	Description of included participants	Age range (years)	*n*
Care provider representatives	Advisors care technology & innovation within aged care institutions. Most participants combined their position with their work as a district nurse.	21–37	8
Potential payer representatives	Representatives of health insurance companies/ long-term care offices with a focus on digital innovation Municipality representatives with a focus on informal care support	35–67	6
Technology provider representatives	CEOs, retailers, and other professionals who work for companies that produce and supply technology to support older adults and their informal caregivers	32–51	6

### Interview- and focus group procedure

The sessions took place at the University of Twente, the participant's workplace, or online. Only the researcher and participant(s) were present. All sessions were led by CW, a male doctoral researcher specialized in dementia care innovation, with experience in conducting qualitative research. All sessions were audio-recorded and lasted approximately 1.5 h. The structure of the sessions followed a topic guide developed by the authors (see Appendix 2), which contained the following elements.

*The case:* We introduced participants to the case by showing a video animation explaining the concept of RM technology in home-based dementia care, how the technology works, and how it might be used by (in)formal caregivers. Subsequently, we presented the value proposition of RM technology for its potential users, which we identified in a previous study among stakeholders in home-based dementia care.^11^ The value proposition for users forms the central element around which business models can be built^32^ and includes a summary of the desired service: A smart and contactless sensor system connected to a platform (app) capable of: A) fall- and wandering detection, B) detection of deviations in self-care (such as eating, drinking, or sleeping), and C) prediction of acute situations (e.g., fall risk prediction).

*Input collection for business models:* To collect input that can be used for business model generation, we used the Business Model Canvas (BMC) of Osterwalder^24^—a popular framework for mapping business models. Participants were asked to provide advice to a hypothetical start-up company that aims to successfully implement RM technology in home-based dementia care. Questions were related to the different elements of the BMC: customer segments (Who are the potential users and payers?), key partners (Which key partners are needed?), channels (How to reach potential users?), customer relationships (How to maintain relationships with customer segments?), key activities (What are key implementation activities?), key resources (Which resources are needed to conduct the key activities?), cost drivers (What are the main cost drivers?), revenue streams (What are suitable funding options?), and value proposition for payers (What are desired outcomes for potential payers?). The BMC elements were questioned in relation to three different scenarios for implementing RM technology in home-based dementia care which can be seen in Figure 1. Those scenarios represent the three main markets for health technology that exist in the Netherlands:^29^

- *Scenario 1 – Consumer market:* The company offers the technology directly to informal caregivers of community-dwelling PwD as a consumer device (business-to-consumer model). The primary focus lies on informal care support.
- *Scenario 2 – Healthcare market:* The company offers the technology to home care organizations where it becomes part of professional care delivery to community-dwelling PwD (business-to-business-to-consumer [B2B2C] model). The district nurse and (if present/optional) informal caregiver are users. The primary focus lies on professional care support.
- *Scenario 3 – Social support market:* The company offers the technology to municipalities which make it available to informal caregivers of community-dwelling PwD (B2B2C model). The primary focus lies on informal care support.

*Discussion of implementation markets:* During the sessions, we also asked participants how feasible they considered the three implementation markets for a company that wants to implement RM technology in home-based dementia care. Particularly, we were interested in learning about potential opportunities and challenges that each implementation market could present to a company.

### Data analysis

The audio recordings of the interviews and focus groups were transcribed verbatim, and content analysis was performed using the software package Atlas.ti 23.

To generate a business model for each of the three implementation scenarios (study aim 1), we used the BMC. Our approach involved the following steps: First, relevant quotes were selected and sorted into the specific BMC element(s) they referred to (customer segments, key partners, channels, customer relationships, key activities, key resources, cost drivers, revenue streams, value proposition for payers). Within each BMC element, the quotes were then further categorized inductively into codes. As a last step, each code was then assigned to one or more of the three scenarios (consumer market, healthcare market, social support market), depending on the scenario(s) the code referred to.

To analyze opportunities and challenges of implementing RM technology via the consumer-, healthcare-, and social support market (study aim 2), relevant quotes were first categorized into one or more of these three scenarios and subcategorized into either (1) opportunities or (2) challenges. Thereafter, selected quotes were further categorized inductively into codes.

To minimize single-researcher bias, a second researcher (AB) independently coded 10% of the data. Points of disagreement were discussed until consensus was reached between CW and AB which resulted in the final coding schemes. The emergence of new codes in the last interview was minimal, suggesting the onset of data saturation.

## Results

### Participant characteristics

Table 1 provides an overview of included participants. A total of 20 participants from across the Netherlands, aged between 21 and 67, took part in the study, with no drop-outs being observed. Each stakeholder group was represented by 6–8 participants. Despite their diverse backgrounds, most participants had in common that digital care innovation played a key role in their jobs.

### Business models for RM technology in home-based dementia care

Our analysis of stakeholder input resulted in different business models for RM technology to support home-based dementia care which specifies how the technology could be introduced and sustain within the Dutch consumer market, healthcare market, and social support market. Illustrative quotes for the codes presented in each business model can be found in Appendix 3.

#### Model 1: Consumer market

**Figure 2.**
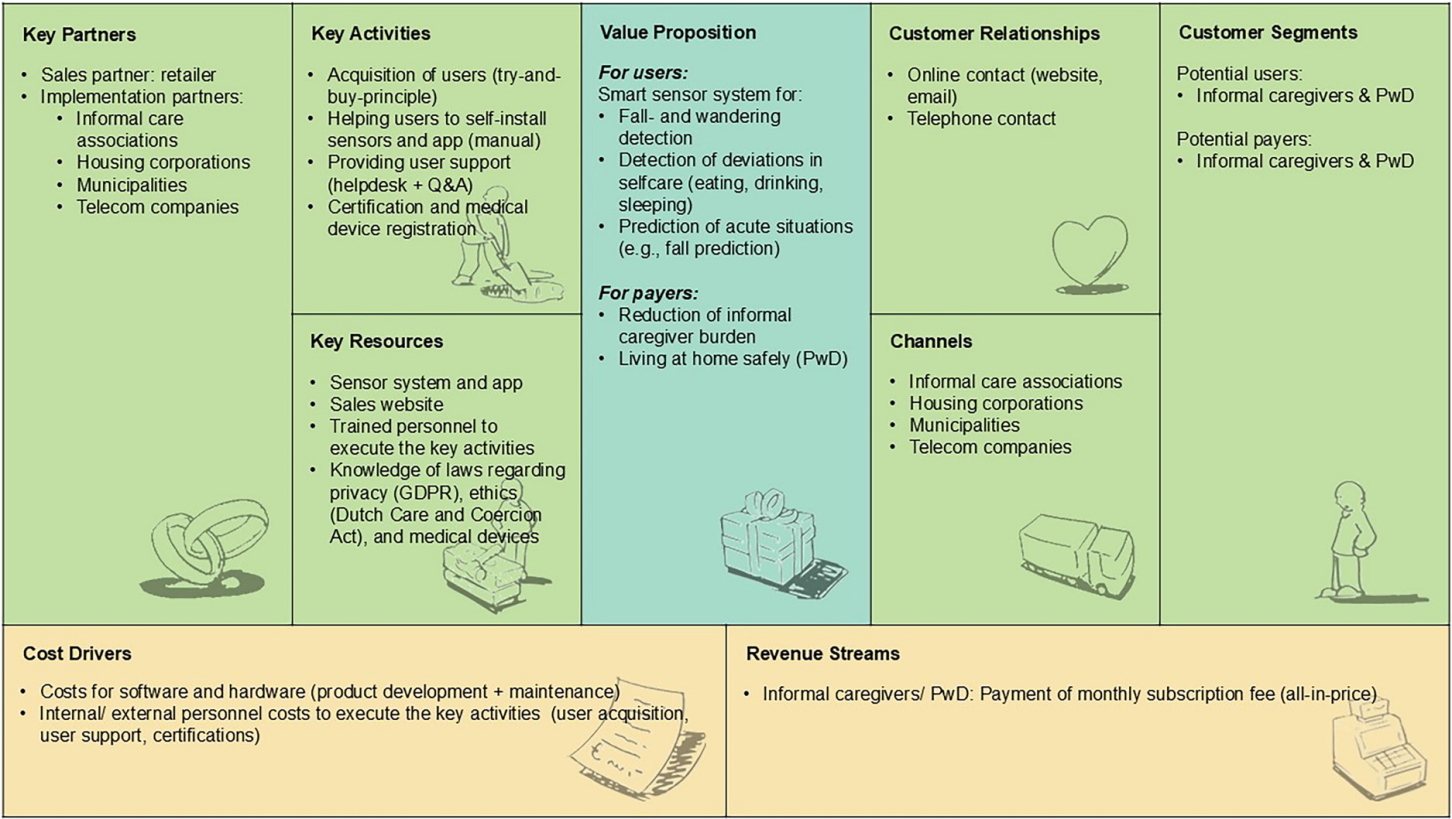
Consumer market business model for RM technology to support home-based dementia care, structured according to the BMC elements.^24^ BMC = Business Model Canvas; RM = remote monitoring.

Figure 2 depicts how RM technology for home-based dementia care could be sustainably implemented via the Dutch consumer market. In this scenario, the technology is directly offered to informal caregivers of community-dwelling PwD as a consumer device (*customer segments*). According to participants, a number of *key partners* are needed to make the business model work: A sales partner (retailer), and implementation partners such as informal care associations, housing corporations, municipalities, and telecom companies, which participants also highlighted as the main *channels* to reach potential users. *Customer relationships* were suggested to be best maintained via online- and telephone contact. Participants recommended several *key activities* to be executed by the company. Those included the acquisition of users (try-and-buy-principle), providing a user manual for self-installation of sensors and the connected app, providing user support (helpdesk + Q&A), certification, and (if applicable) medical device registration for the technology. *Key resources* to conduct those activities include the sensor system with connected app, a sales website, trained personnel, and knowledge of laws regarding privacy, ethics, and medical devices. These resources were also seen as the main *cost drivers* of the business model. According to participants, the model could ultimately be financed through monthly subscription fees paid by informal caregivers/PwD (*revenue streams*). This is under the condition that the technology reduces informal caregiver burden and enables PwD to live at home safely (*value proposition for payers*).

#### Model 2: Healthcare market

**Figure 3.**
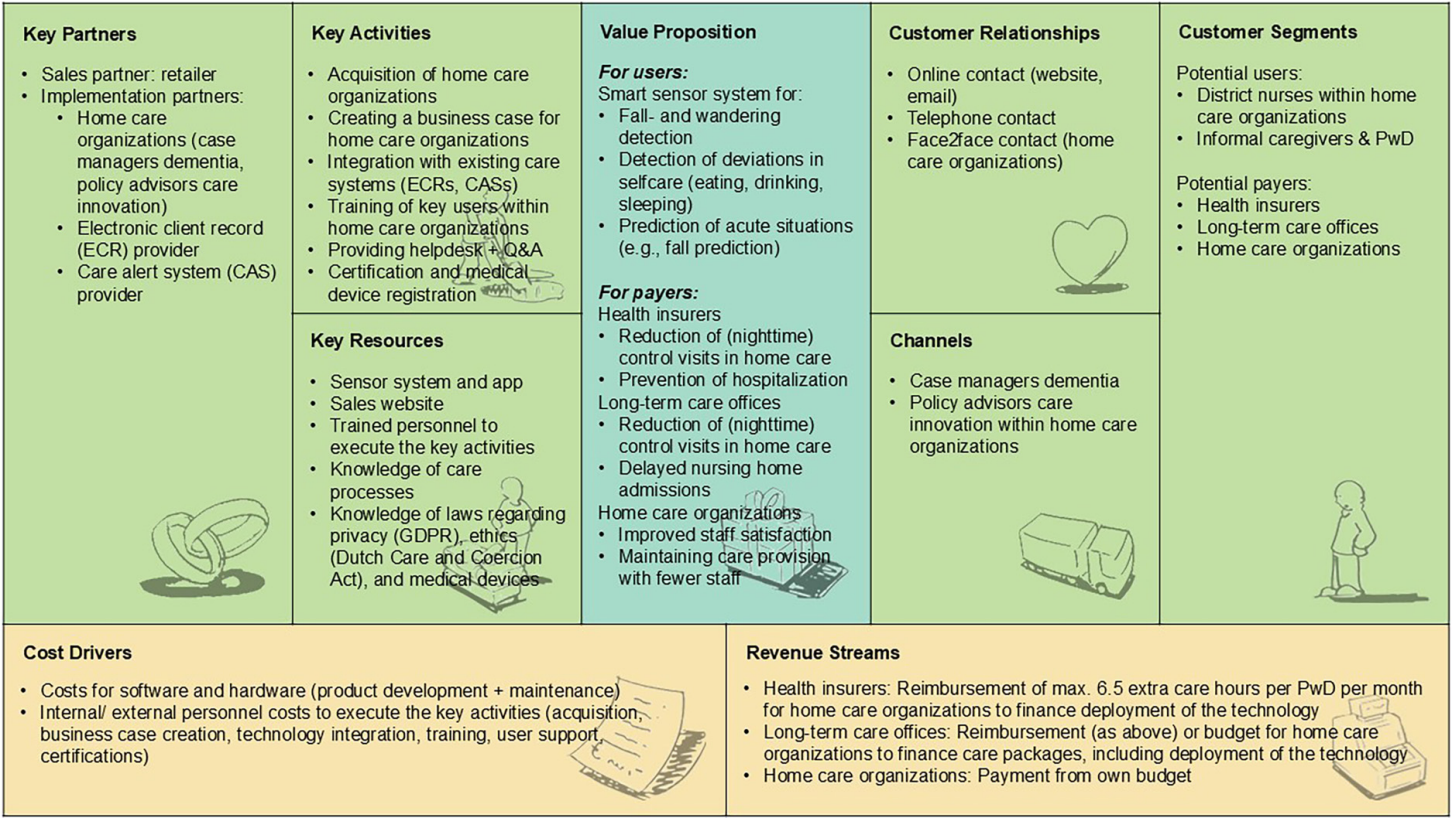
Healthcare market business model for RM technology to support home-based dementia care, structured according to the BMC elements.^24^ BMC = Business Model Canvas; RM = remote monitoring.

Figure 3 depicts how RM technology for home-based dementia care could be sustainably implemented via the Dutch healthcare market. In this scenario, RM technology is offered to home care organizations (*customer segments*) and becomes part of professional care delivery to community-dwelling PwD. The district nurse and (if present/optional) informal caregiver are users. According to participants, a number of key partners are needed to make the business model work. These include a sales partner (retailer), and providers of existing digital infrastructures in home care (electronic client records [ECRs], care alert systems [CASs]). Additional key partners are case managers dementia and policy advisors care innovation within home care organizations, who can also play a role in reaching potential users (channels). Customer relationships were suggested to be maintained via online, telephone, and face-to-face contact. Participants highlighted several key activities that the company must undertake, including the acquisition of home care organizations using an attractive business case, the integration of the technology with existing digital infrastructures (ECRs, CASs), training of key users within home care organizations, providing user support (helpdesk + Q&A), certification, and (if applicable) medical device registration for the technology. Key resources to conduct those activities include the sensor system with connected app, a sales website, trained personnel, and knowledge about care processes, privacy law, ethics, and medical devices. These resources were also considered the main cost drivers of the business model. According to participants, the model can ultimately be financed via three options (revenue streams): (1) Health insurers that reimburse additional care hours per PwD per month, allowing home care organizations to finance the deployment of the technology (subscriptions, implementation costs), (2) Long-term care offices that provide the same reimbursement option or an integral budget for home care organizations to finance care packages for PwD, including deployment of the technology, or (3) Home care organizations that finance the technology from own budget. Participants stressed that each of those financing options requires the technology to deliver different outcomes (value proposition for payers). Health insurers expect cost savings through a reduction of (nighttime) control visits in home care and the prevention of hospitalizations. Long-term care offices seek cost savings as well, through fewer (nighttime) home care visits, along with delayed nursing home admissions. Lastly, home care organizations expect the technology to improve staff satisfaction and to help maintain care provision with fewer staff (due to growing personnel shortages).

#### Model 3: Social support market

**Figure 4.**
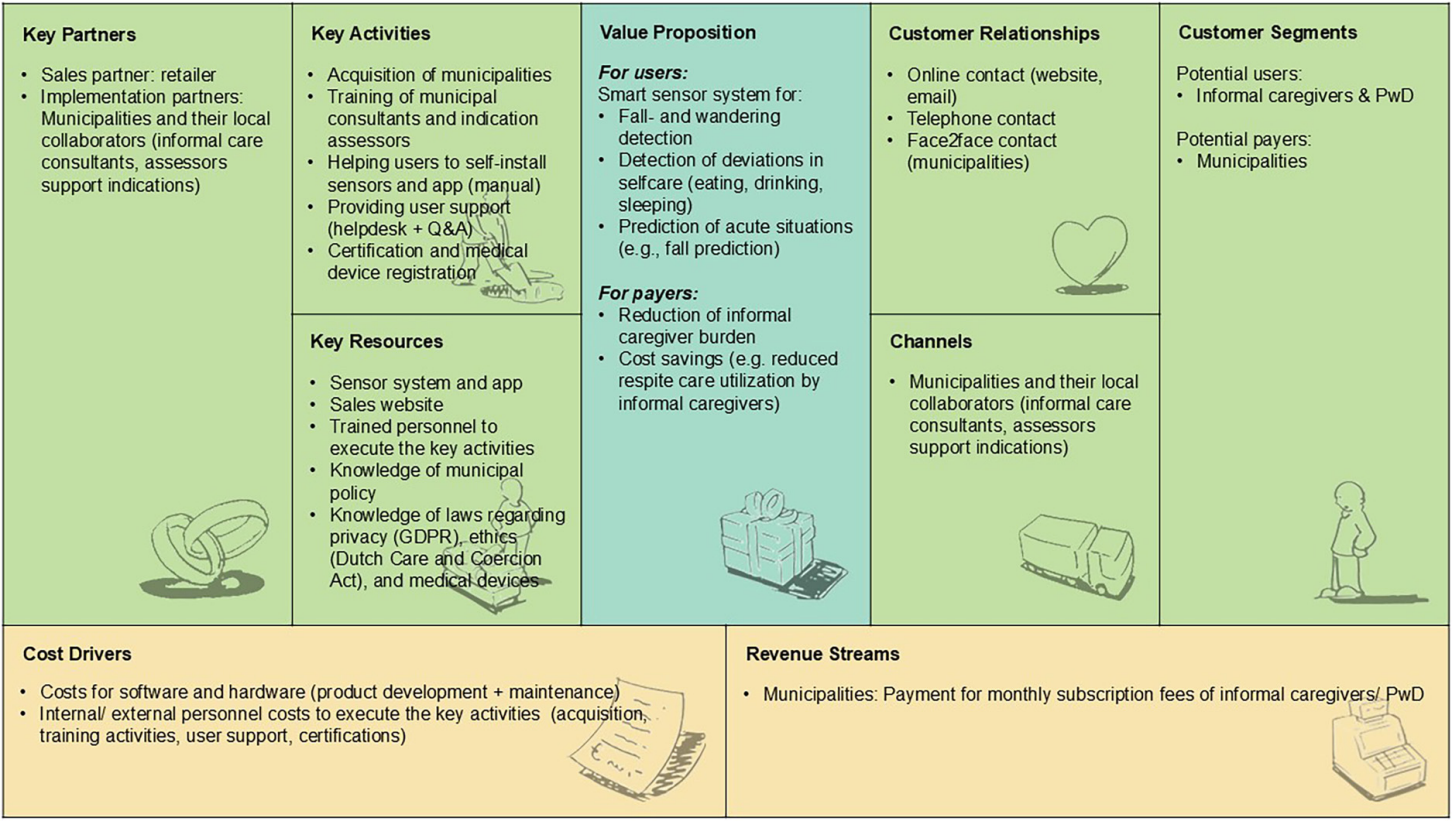
Social support market business model for RM technology to support home-based dementia care, structured according to the BMC elements.^24^ BMC = Business Model Canvas; RM = remote monitoring.

Figure 4 depicts how RM technology for home-based dementia care could be sustainably implemented via the Dutch social support market. In this scenario, the technology is offered to municipalities which make it available to informal caregivers of community-dwelling PwD (*customer segments*). According to participants, a number of *key partners* are required to make the business model work. These included a sales partner (retailer), and implementation partners including municipalities and their collaborators (informal care consultants, assessors support indications), who were also suggested as the main *channels* to reach informal caregivers of community-dwelling PwD. Customer relationships were suggested to be maintained via online, telephone, and face-to-face contact. Participants highlighted several key activities to be executed by the company, including the acquisition of municipalities, training of municipal consultants, and support indication assessors in matching the technology to the right users, providing a user manual for self-installation of sensors and app, providing user support (helpdesk + Q&A), certification, and (if applicable) medical device registration for the technology. Key resources to execute those activities include the sensor system with connected app, a sales website, trained personnel, knowledge about municipal policy and laws regarding privacy, ethics, and medical devices. These resources were also seen as the main cost drivers of the business model. Participants noted that the model can ultimately be financed (revenue streams) by municipalities who pay for monthly subscription fees of informal caregivers/PwD. This is under the condition that the technology reduces informal caregiver burden and creates cost savings for municipalities (e.g., through reduced respite care utilization by informal caregivers) (value proposition for payers).

### Opportunities and challenges of implementing RM technology via different markets

Our analysis of the stakeholder sessions identified a number of entrepreneurial opportunities and challenges of implementing RM technology for home-based dementia care via the Dutch consumer-, healthcare-, and social support market. An overview of topics per market can be found in Table 2. Most topics came forward from two or all three stakeholder groups (care provider representatives, potential payer representatives, and technology provider representatives).

**Table 2.** Overview of opportunities and challenges per implementation market.

Topics	Consumer market	Healthcare market	Social support market
Opportunities			
- Early deployment of technology possible	C, P		C, P
- Aligned with growing shift toward informal care	C, P, T		C, P, T
- Fast way to market	P, T		
- Potentially higher user acceptance		T	
- Easy access to potential user groups		C, P	
Challenges			
- Target group hard to reach	P, T		
- Possibly low willingness to pay	C, P, T		
- High personnel costs per customer	T		
- Delayed introduction of the technology in the disease pathway		C, P	
- Way to structural reimbursement long and complex		C, P, T	
- Upscaling difficult			C, P, T
- Unclear financial responsibility and incentive			C, P

*Note.* C = Care provider representatives; P = Potential payer representatives; T = Technology provider representatives. A topic was linked to a specific stakeholder group if it came forward by at least one participant from that group.

#### Consumer market opportunities

##### Early deployment of technology possible

Participants mentioned that, when implemented via the consumer market, RM technology for PwD can be deployed at an early disease stage already, even without a dementia diagnosis, regardless of whether professional home care is involved in the care. This creates a large potential target group. Given the trend that indications for home care are increasingly delayed due to shortages of staff, RM technology can help to bridge the gap and provide support to PwD and their informal caregivers early-on.

Well, what I don’t like is that using technology always starts with a healthcare need, whereas you would actually want to start earlier. […] You want a certain level of health and to have control over it yourself. And I think it's similar for people in the early stages of dementia. (health insurer representative)

##### Aligned with growing shift toward informal care

Participants noted that RM technology, which primarily targets informal caregivers of PwD in the consumer market, would align well with future trends. As the reliance on informal care continues to grow due to a worsening shortage of professional caregivers, the responsibility for nonmedical tasks (e.g., check-ins, daily support) will increasingly shift from district nursing to informal caregivers. This shift creates a growing demand for supportive consumer devices, some of which are already being used by informal caregivers of PwD today (e.g., Google Home and smartwatches).

I think we’re increasingly moving toward the consumer market scenario […] because we are scaling everything back and ask the question: What can you do yourself? What can your family do? What can assistive devices do? And only then comes professional home care. (advisor care technology within aged care institution)

##### Fast way to market

Participants considered the consumer market as less complex and therefore a potentially fast way to implement RM technology for home-based dementia care. Companies would not have to deal with complex evaluation procedures and requirements of care financing bodies. This can be particularly advantageous for companies whose investors expect timely results.

If I reason from a business perspective, it's easiest for me to bring the technology into the consumer market because it is the least complex. (age tech company representative)

#### Consumer market challenges

##### Target group hard to reach

Participants considered it challenging to directly reach out to informal caregivers of community-dwelling PwD in the consumer market, as this target group is generally unregistered. Strategic partners (such as informal care associations or housing corporations) would be needed to get in touch with the target group and successfully implement RM technology.

Reaching informal caregivers through a direct line—that is something I would find quite challenging. (health insurer representative)

##### Possibly low willingness to pay

Successful implementation of RM technology via the consumer market might be hindered by a low willingness to pay by informal caregivers of PwD. Participants noted that, in the Netherlands, citizens are relatively accustomed to having care arranged for them. Consequently, users would probably expect the technology to be financed by their health insurer, long-term care office, or municipality.

In the Dutch healthcare system, there is always room for people to purchase and use care technologies themselves. However, when a healthcare system has set up a lot, for instance via the Wmo [Social Support Act] or Wlz [Long-term Care Act], people automatically look first at what is offered by organizations within those domains. (health insurer representative)

##### High personnel costs per customer

According to participants, a scenario in which RM technology is offered to individual users bears the risk of high personnel costs within the company (e.g., acquisition, sales). Offering the technology to an organization (e.g., a home care organization) that purchases the technology for a range of its clients would lower this risk.

Suppose you employ people, then you will not earn back the costs you have to make to implement the system for one person. (age tech retailer representative)

#### Healthcare market opportunities

##### Potentially higher user acceptance

Participants considered the chance of acceptance of RM technology among PwD higher when the technology is implemented via professional home care. In this scenario, the technology serves a professional purpose. In addition, district nurses are seen as reliable and trustable initiators to discuss the deployment of RM technology, together with PwD and their informal caregivers.

I think it is good if the care professional plays a role in the conversation. […] I think that scenarios one [consumer market] and three [social support market] have to be initiated by the informal caregiver and this sometimes leads to a difficult conversation, in which the district nurse can be of great help. (CEO age tech company)

##### Easy access to potential user groups

Implementing RM technology via home care organizations offers the advantage of relatively easy access to both potential user groups (district nurses and informal caregivers of community-dwelling PwD). Reaching out to users via the consumer market or via municipalities, on the other hand, is more challenging.

For me, this can only be done in collaboration with the home care organization, so option two [healthcare market] is the most realistic option for me. (municipality representative)

#### Healthcare market challenges

##### Delayed introduction of the technology in the disease pathway

When implemented through the healthcare market, RM technology is only used for PwD who have a home care indication, which is typically the case when a PwD's care needs become more complex along with the disease pathway. Excluding PwD in the early stages of the condition not only reduces the target group in size but may also result in delayed implementation, missing out on the potential preventive benefits of the technology (e.g., delaying the need for district nursing).

If someone already receives home care, then you are actually already much too late [with implementing RM technology]. You also want to see what we can do for prevention. (advisor care technology within aged care institution)

##### Way to structural reimbursement long and complex

The road to securing structural reimbursement for RM technology in home-based dementia care through health insurers and long-term care offices is lengthy, involving pilots to demonstrate added value. For instance, demonstrating a possible delay in nursing home admission requires research over an extended period. Additionally, reimbursement via health insurers can only occur if the technology replaces care (for instance, by reducing nighttime visits or preventing care in other settings such as hospitals). At present, it is unclear whether RM technology will decrease or increase healthcare utilization, due to responses to system alerts. “Wrong pocket” issues also complicate financing, particularly when costs and benefits of the technology do not fall within the same domain (e.g., when health insurers bear costs but long-term care offices reap benefits from delayed nursing home admissions).

It's almost unavoidable to conduct a pilot with a number of patients and home care institutions to demonstrate that the outcomes result in delayed care needs. And that, of course, is challenging because it takes a lot of time. Especially if you believe this [the technology] could lead to people entering a nursing home later—you’d need to show that over a longer period. (health insurer representative)

#### Social support market opportunities

##### Early deployment of technology possible

Similar to the consumer market, participants noted that RM technology can also be deployed at an early disease stage already when implemented via municipalities. In this case, the technology can benefit informal caregivers of community-dwelling PwD, regardless of whether or not professional home care is involved in the care.

I would like to say to providers of this technology: How could you reach me as a daughter caring for her father with dementia? To say hey, this is interesting, we are already going to install this in our house. I think that's a really interesting angle. And it's not about keeping it away from the health insurers, not at all. (health insurer representative)

##### Aligned with growing shift toward informal care

Participants noted that, similar to the consumer market, the implementation of RM technology via municipalities would be in line with the increasing shift from district nursing toward informal care. Municipalities are responsible for informal care support. As the reliance on informal caregivers continues to grow, so does the need for assistive devices such as RM technology.

I would say focus on the large group, and that is the informal caregivers, that will soon be the large group. We have to serve them first because that is where the problems are, despite the fact that the problems are already in professional care. But the informal caregiver group is going to grow the fastest. (CTO age tech company)

#### Social support market challenges

##### Upscaling difficult

A local approach for implementing RM technology via municipalities can work well. However, each municipality organizes support for PwD and their informal caregivers in its own unique way, often involving different local parties. In some cases, municipalities provide support directly; in others, it is arranged through care and welfare organizations or via personal budgets allocated to citizens. These differences between municipalities create significant barriers to scaling up RM technology to a national level.

Municipalities are allowed to do everything in their own way. Municipality X can do it very differently than the neighbouring municipality Y. (advisor care technology within aged care institution)

I think that as a technology provider you do not want to have to deal with seven different financing systems in three different municipalities. (health insurer representative)

##### Unclear financial responsibility and incentive

Municipalities can decide for themselves how they provide support for PwD and their informal caregivers under the Social Support Act (Wmo). However, digital care solutions, including RM technology, are relatively new to municipalities, leading to reluctance in funding these innovations. Furthermore, it is unclear whether municipalities would continue to finance the technology once a PwD has received an indication for professional home care. In such cases, it is more likely that the health insurer or long-term care office would take on the financial responsibility. Additionally, wrong pocket problems can complicate financing via municipalities. This refers to situations where municipalities invest in RM technology, but the financial benefits (such as delayed nursing home admissions) flow to the long-term care sector instead.

The Wmo [municipal support] is still very much behind in terms of its commitment to technology. (advisor care technology within aged care institution)

Everything the municipality invests in benefits the long-term care sector. (municipality representative)

## Discussion

### Principal findings

Our study resulted in business models for RM technology to support home-based dementia care, which can guide implementation within three different markets: the Dutch consumer-, healthcare-, and social support market. Additionally, our study identified possible entrepreneurial opportunities and challenges of implementing RM technology for home-based dementia care via those three markets. The results of our study respond to current literature reviews on the implementation of assistive technologies in dementia care, which highlight the need for more research on how viable business models in this field can operate^33^ and aspects of the wider implementation context that technology providers, in particular, need to consider.^34^ Our study reduces this knowledge gap in the context of RM technology for home-based dementia care.

The market-specific business models generated in this study mainly differ regarding the customer segments which are targeted, the funding structures because of different potential payers, and the infrastructure needed for implementation. All three models specify which outcomes of RM technology are expected by potential payers. Our study shows that these outcomes are different per implementation market. While the implementation of RM technology via the healthcare and social support market requires the technology to primarily create cost savings (e.g., reduction of home care visits, delayed nursing home admissions, reduced respite care utilization), this is less relevant for the consumer market in which outcomes such as a reduced caregiver burden stay central.

In the current study, we generated three distinct business models for RM technology which, in principle, could be applied chronologically along a home-dwelling PwD's care journey. In the Netherlands, home-dwelling PwD often pass through different care sectors: (1) social support funded by municipalities, (2) regular home care funded by health insurers, and (3) intensive home care funded by long-term care offices.^35^ Informal caregivers (if present) often provide support throughout the entire journey. In that regard, companies could, for instance, apply the consumer market model or the social support market model targeting PwD in the early stages of the disease when no professional home care is yet involved, and apply the healthcare market model as soon as a PwD receives an indication for professional home care. While this could ensure the continuity of technology deployment, questions remain whether it is feasible for a company to target a diverse group of users across various care sectors. Previous research has highlighted that digital health technologies which target multiple fragmented care funding schemes currently encounter complex issues in reaching national scale.^36^ In the future, payer organizations (such as municipalities, health insurers, and long-term care offices) will have to work together on sector-overarching funding options to enable PwD and their caregivers to use RM technology and other technologies along the full care journey. Nevertheless, the business models generated in the current study could help developers of RM technology in home-based dementia care to design new or refine existing business models that are attuned to its specific market. It should be noted, however, that business models are never fixed: Chances are that they need to be updated constantly, in line with, for example, new policies and changes in funding structures.^37^

Our study shows that each implementation market brings a number of entrepreneurial opportunities and challenges for implementing RM technology in home-based dementia care. We noted that most of these findings are, however, not limited to RM technology alone. For instance, healthcare market opportunities (such as easier access to PwD and their caregivers) and healthcare market challenges (such as a long and complex way to structural reimbursement) are likely to be applicable to other technologies for PwD and their caregivers as well. Comparing the key findings for each market, we can conclude the following: The consumer market appears to be especially future-oriented in terms of its alignment with the growing shift toward informal care, but the target group (home-dwelling PwD and their informal caregivers) is currently difficult to reach via this market. The healthcare market offers good opportunities for scaling up RM technology, and the target group is easier to reach via home care organizations, but this market is also complex in terms of securing structural funding for RM technology. The social support market shares similar opportunities as the consumer market, but scaling up RM technology is challenging because each municipality organizes care and support in a different way. Ultimately, the choice for an implementation market for RM technology in home-based dementia care will depend on the developers themselves. However, our findings can help in selecting the most suitable market.

### Strengths and limitations

A strength of this study lies in the use of a well-established framework to generate business models: The BMC.^24^ The BMC allowed us to systematically map the key components of a company that are required to sustainably implement RM technology in different markets. We used the BMC in the original way by focussing on companies, however, the BMC can also be applied successfully for nonprofit organizations such as care organizations,^26^ as has been shown in a previous study of ours.^11^ Furthermore, we generated the business models and implementation considerations per market in a stakeholder-driven way, by consulting various stakeholders who play a key role in implementing RM technology for home-based dementia care. This ensured that our results would reflect diverse stakeholder perspectives, instead of only the perspectives of business professionals.

Our study does, however, not come without limitations. Our results have been obtained within the context of the Dutch (dementia) care system, which might limit their transferability to the context of other countries. Each country's implementation context is unique due to variances in how care and support is organized and financed. However, despite differences in care systems, we believe that our proposed business models and market-specific considerations for implementing RM technology are relevant in an international context too as they can serve as a basis to further build upon. This mainly pertains to the results concerning the consumer- and healthcare market, whereas the social support market (municipality support) is fairly specific to the Netherlands. Furthermore, due to the time-consuming nature of our approach, we did not include an additional feedback round to check how far participants felt their views were represented in the final results. This could have enhanced the robustness of the findings. Additionally, we cannot rule out that our sample might overrepresent participants who were already enthusiastic about the topic. At the same time, however, our participants dared to be critical too, by also expressing pessimistic views on RM technology implementation. Lastly, for generating the business models, we relied on interview- and focus group data only, whereas it can be beneficial to use multiple methods, including desk research as well.^38^ In that way, the risk of missing out on relevant input (e.g., national legislations, funder requirements) can be minimized.

### Future research

The business models generated in this study can guide future implementation of RM technology in home-based dementia care. We invite researchers and developers from other countries to adapt them according to preconditions that are specific to the national dementia care system in place and to further optimize our business models. This optimization could be done using an in-depth SWOT analysis (strengths, weaknesses, opportunities, threats) involving relevant stakeholders, for which Osterwalder et al.^24^ have created a practical guide.

Ultimately, upscaling RM technology in home-based dementia care requires not only a viable business model but also a viable business case for every party involved (e.g., the company, home care organizations, municipalities). Business cases build upon business models and provide deeper insight into the balance between benefits and costs, and thereby the economic viability of a technology.^39^ Often, (prospective) business cases for RM technology and other home care technologies are requested in negotiations between care organizations and health insurers. Future research should focus on creating and openly publishing these business cases so that care organizations can adapt them to their own organization. In cases where benefits and costs of the technology are expected to fall across multiple stakeholders, a societal business case based on a Social Return On Investment (SROI) analysis^40,41^ could provide insight. This method can ultimately form an instrument to discuss the financing with all potential payers or decision-makers involved, based on the expected societal impact of the technology.^41^

## Conclusions

Our findings contribute to the limited body of research on the entrepreneurial side of implementing dementia care technology. Focussing on RM technology for home-based dementia care, our results provide guidance for implementation across three different markets: the consumer-, healthcare-, and social support market. For each of these markets, we propose a distinct business model which could aid developers in refining implementation strategies. Furthermore, our study shows that each implementation market brings specific points of attention that need to be considered. Topics dealt with, for instance, reaching potential user groups, time to market, the complexity of structural reimbursement, scalability, and alignment with future care trends. These insights could help developers of RM technology and other technologies for home-based dementia care to select a suitable implementation market. Lastly, our study might inspire other researchers aiming to develop business models for dementia care technology in a stakeholder-driven way.

## Supplemental Material

sj-pdf-1-dhj-10.1177_20552076251331825 - Supplemental material for Smart monitoring technology to support home-based dementia care: Market-specific business model development and implementation considerations in the Netherlands Supplemental material, sj-pdf-1-dhj-10.1177_20552076251331825 for Smart monitoring technology to support home-based dementia care: Market-specific business model development and implementation considerations in the Netherlands by Christian Wrede, Annemarie Braakman-Jansen and Lisette van Gemert-Pijnen in DIGITAL HEALTH

sj-pdf-2-dhj-10.1177_20552076251331825 - Supplemental material for Smart monitoring technology to support home-based dementia care: Market-specific business model development and implementation considerations in the Netherlands Supplemental material, sj-pdf-2-dhj-10.1177_20552076251331825 for Smart monitoring technology to support home-based dementia care: Market-specific business model development and implementation considerations in the Netherlands by Christian Wrede, Annemarie Braakman-Jansen and Lisette van Gemert-Pijnen in DIGITAL HEALTH

sj-pdf-3-dhj-10.1177_20552076251331825 - Supplemental material for Smart monitoring technology to support home-based dementia care: Market-specific business model development and implementation considerations in the Netherlands Supplemental material, sj-pdf-3-dhj-10.1177_20552076251331825 for Smart monitoring technology to support home-based dementia care: Market-specific business model development and implementation considerations in the Netherlands by Christian Wrede, Annemarie Braakman-Jansen and Lisette van Gemert-Pijnen in DIGITAL HEALTH
