# Supplementary material for: Smart monitoring technology to support home-based dementia care: Market-specific business model development and implementation considerations in the Netherlands
Source: Digit Health. 2025 Mar 28;11:20552076251331825. doi: 10.1177/20552076251331825 (PMC11951899; doi:10.1177/20552076251331825)

Appendix 2: Interview- and focus group guide

Topic	Questions/ explanations
**Start**	Discuss with participant(s):<br>- Introduction of researcher<br>- Purpose, content and structure of interview/ focus group<br>- Participant is free to skip questions he/she cannot answer<br>- Participants can ask questions at any time<br>- Audio recording<br>- Written/ digital informed consent<br>- Clarification of any remaining questions
**Background questions**	Background questionnaire (age, current function, employer, number of years of work experience in current function)
**The case**	Presentation of video animation explaining the concept of RM technology in home-based dementia care (a selection of screenshots can be found at the end of the document, p. 3)<br><br>Presentation of the value proposition of RM technology for its potential users (the desired service):<br>A smart and contactless sensor system connected to a platform (app) capable of:<br>a) fall- and wandering detection,<br>b) detection of deviations in self-care (such as eating, drinking or sleeping) and<br>c) prediction of acute situations (e.g., fall risk prediction)
**Explanation of market scenarios**	In front of you, you can see descriptions of different potential implementation markets for RM technology in home-based dementia care. Let's walk through them:<br>1) Consumer market scenario: The company offers the technology directly to informal caregivers of community-dwelling PwD as a consumer device (business-to-consumer (B2C) model). The primary focus lies on informal care support.<br>2) Healthcare market scenario: The company offers the technology to home care organizations where it becomes part of professional care delivery to community-dwelling PwD (business-to-business-to-consumer (B2B2C) model). The district nurse and (if present/optional) informal caregiver are users. The primary focus lies on professional care support.<br>3) Social support market scenario: The company offers the technology to municipalities that make it available to informal caregivers of community-dwelling PwD (business-to-business-to-consumer (B2B2C) model). The primary focus lies on informal care support.
**Instruction for BMC input collection**	For each of the three scenarios, we would like to create a business model which can facilitate sustainable implementation of RM technology in home-based dementia care. To do so, we use the Business Model Canvas (BMC) which you can see in front of you. The BMC consists of different elements which we will go through together. For each of those elements, please imagine you would have to give advice to a hypothetical start-up company who aims to successfully implement RM technology in home-based dementia care.

**Customer segments**	For scenario 1, 2, and 3:<br>- Who are the potential users and payers of RM technology in home-based dementia care?<br>- Would you like to change or add any users/payers you see in the scenarios?
**Key partners**	For scenario 1, 2, and 3:<br>- Which key partners are essential for a company aiming to successfully implement RM technology in home-based dementia care?
**Channels**	For scenario 1, 2, and 3:<br>- How could a company reach the potential users best?
**Customer relationships**	For scenario 1, 2, and 3:<br>- How could a company maintain a relationship with its users (and eventually payers)?
**Key activities**	For scenario 1, 2, and 3:<br>- What are the most important activities that a company would need to execute to successfully implement RM technology in home-based dementia care?
**Key resources**	For scenario 1, 2, and 3:<br>- Which resources (e.g. human, intellectual, physical) are needed to conduct the key activities?
**Cost drivers**	For scenario 1, 2, and 3:<br>- What would be the main cost drivers for a company aiming to implement RM technology?
**Revenue streams**	For scenario 1, 2, and 3<br>- What are suitable direct and indirect funding sources that could be used to create revenue?<br>- How do those funding schemes work?
**Value proposition for payers**	For scenario 1, 2, and 3<br>- Which outcomes of RM technology would potential payers like to see?<br>- How should those outcomes be demonstrated?
**Considerations for different market scenarios**	In general:<br>- How feasible do you consider the three market scenarios for a company who wants to successfully implement RM technology in home-based dementia care?<br>- Can you imagine possible opportunities or challenges that each implementation market could pose to a company? If yes, please explain.
**End**	Are there any aspects that we have not yet discussed in the previous sections that you would like to share?<br>Thank you very much for your time and valuable help.

Selection of screenshots of the video animation

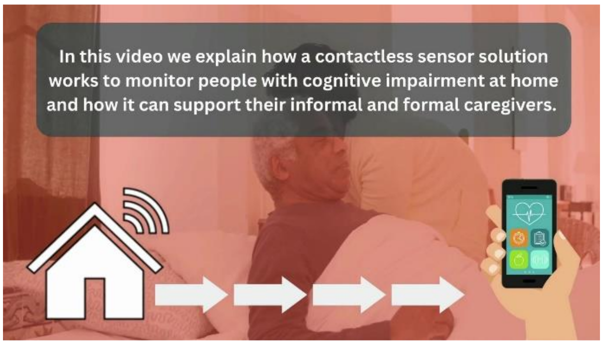

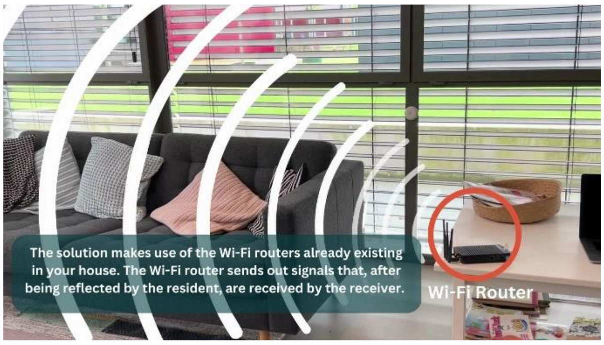

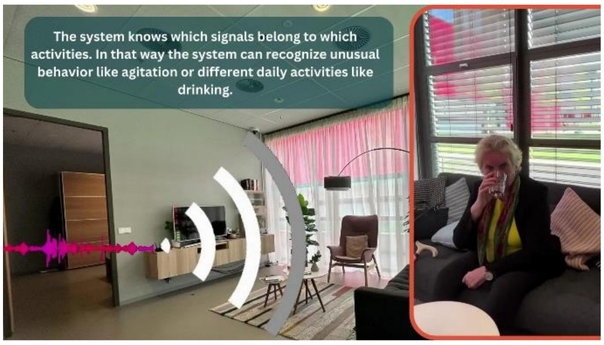

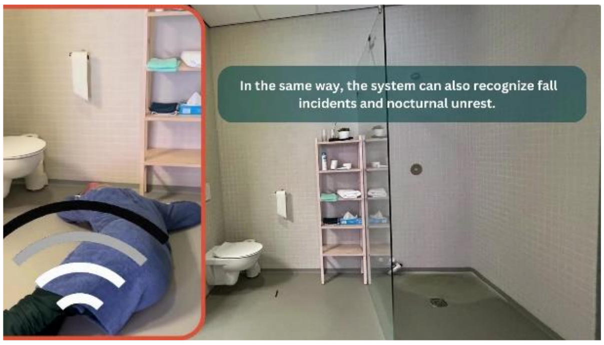

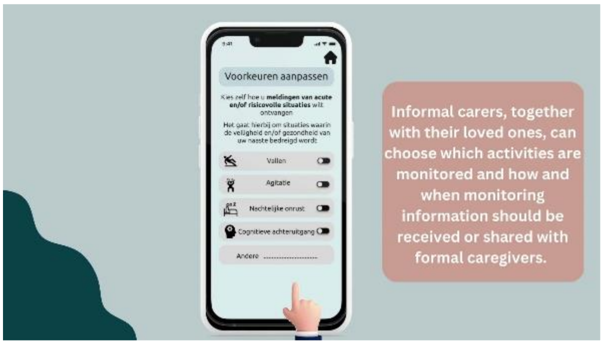

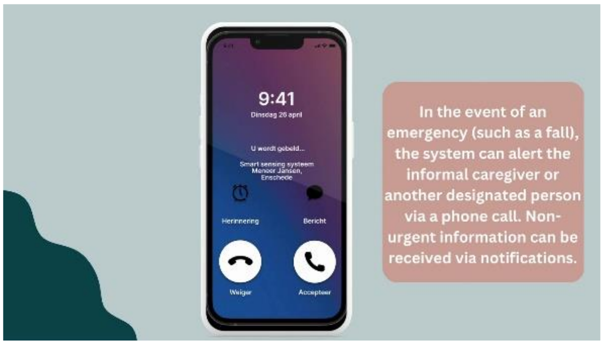

Supplement: sj-pdf-2-dhj-10.1177_20552076251331825 - Supplemental material for Smart monitoring technology to support home-based dementia care: Market-specific business model development and implementation considerations in the Netherlands [file sj-pdf-2-dhj-10.1177_20552076251331825.pdf]
