# Supplementary material for: Smart monitoring technology to support home-based dementia care: Market-specific business model development and implementation considerations in the Netherlands
Source: Digit Health. 2025 Mar 28;11:20552076251331825. doi: 10.1177/20552076251331825 (PMC11951899; doi:10.1177/20552076251331825)
Supplement: sj-pdf-3-dhj-10.1177_20552076251331825 - Supplemental material for Smart monitoring technology to support home-based dementia care: Market-specific business model development and implementation considerations in the Netherlands [file sj-pdf-3-dhj-10.1177_20552076251331825.pdf]

Appendix 3: Codes and illustrative quotes for business models

Business model 1: Consumer market

**BMC element**	**Code**	**Illustrative quote(s) in original language (Dutch)**
**Customer segments**<br>(Who are the potential users and payers?	Potential users:<br>Informal caregivers & PwD	N.a. (predefined by scenario)
	Potential payers:<br>Informal caregivers & PwD	N.a. (predefined by scenario)
**Key partners**<br>(Which key partners are needed?)	Sales partner: Retailer	Session 5 - Interview 07.11.2023: *’’Ja maar ik bedoel ook echt even een retailer als in de winkels die je in de straat tegenkomt. Dat kan dus ook bijvoorbeeld een online winkel zijn.’’* (health insurer representative)
	Implementation partner:<br>Informal care associations	Session 13 - Interview 30.11.2023: *’’Ik ken niet helemaal de wereld van de mantelzorg maar er zijn wat mantelzorgverenigingen en die hebben denk ik best wel mogelijkheid om mantelzorg in Nederland te sturen. Zeker als zij achter zo'n initiatief staan dan heb je wel mogelijkheden.’’* (health insurer representative)
	Implementation partner:<br>Housing corporations	Session 9 - Interview 22.11.2023*: ’’Maar er is nog een partij die ik heel erg van belang vind hier en dat zijn woningcorporaties. En woningcorporaties hebben het belang om mensen een passende woning aan te bieden tegen een betaalbare huur en die hebben daar ook een rol in.’’* (municipality representative)
	Implementation partner:<br>Municipalities	Session 5 - Interview 07.11.2023: *’’Wat je eigenlijk ook in die gebruiksscenario's ziet is dat een aantal van die segmenten die je nu gedefinieerd hebt, die zou je ook als kanaal kunnen zien. De thuiszorginstelling zou bijvoorbeeld je distributiekanaal kunnen zijn om je propositie bij de mantelzorgers en patiënten te krijgen. Hetzelfde geldt voor gemeenten.’’* (health insurer representative)
	Implementation partner:<br>Telecom companies	Session 8 - Interview 20.11.2023: *’’Ik zou het heel anders doen, bijvoorbeeld naar KPN of Ziggo gaan en zeggen: iedereen die daar een wifi abonnement afsluit, jij krijgt dit [het sensor systeem] cadeau voor jou, heb je een ouder thuis, dan krijg je dit cadeau. En nou, dat op die manier vinden wij als KPN het bijvoorbeeld belangrijk dat de mensen langer thuis kunnen wonen, zoiets zou ik eerder doen, zo'n soort model.’’* (age tech retailer representative)
**Channels**<br>(How to reach potential users?)	Informal care associations	Session 13 - Interview 30.11.2023: *’’Ik ken niet helemaal de wereld van de mantelzorg maar er zijn wat mantelzorgverenigingen en die hebben denk ik best wel mogelijkheid om mantelzorg in Nederland te sturen. Zeker als zij achter zo'n initiatief staan dan heb wel mogelijkheden.’’* (health insurer representative)

	Housing corporations	Session 9 - Interview 22.11.2023: *''Maar er is nog een partij die ik heel erg van belang vind hier en dat zijn woningcorporaties. En woningcorporaties hebben het belang om mensen een passende woning aan te bieden tegen een betaalbare huur en die hebben daar ook een rol in.''* (municipality representative)
	Municipalities	Session 5 - Interview 07.11.2023: *''Wat je eigenlijk ook in die gebruiksscenario's ziet is dat een aantal van die segmenten die je nu gedefinieerd hebt, die zou je ook als kanaal kunnen zien. De thuiszorginstelling zou bijvoorbeeld je distributiekanaal kunnen zijn om je propositie bij bij de mantelzorgers en patiënten te krijgen. Hetzelfde geldt voor gemeenten.''* (health insurer representative)
	Telecom companies	Session 8 - Interview 20.11.2023: *''Ik zou het heel anders doen, bijvoorbeeld naar KPN of Ziggo gaan en zeggen: iedereen die daar een wifi abonnement afsluit, jij krijgt dit [het sensor systeem] cadeau voor jou, heb je een ouder thuis, dan krijg je dit cadeau. En nou, dat op die manier vinden wij als KPN het bijvoorbeeld belangrijk dat de mensen langer thuis kunnen wonen, zoiets zou ik eerder doen, zo'n soort model.''* (age tech retailer representative)
**Customer relationships**<br>(How to maintain relationships with customer segments?)	Online contact (website, email)	Session 4 - Interview 01.11.2023: *''Uitleg geven hoe het werkt via instructies, video's, duidelijke website met veelgestelde vragen, handleidingen.''* (advisor care technology within aged care institution)
	Telephone contact	Session 2 - Interview 24.10.2023: *''Wat wij ook vaak doen is wij informeren de mantelzorger over onderhoud en dit soort dingen via whatsapp. En dat is ook hetzelfde nummer waarop zij een hulpverzoek doen en daarmee houden wij dus ook ons gezicht en onze namen en ons contact heel dichtbij bij de klant.''* (age tech company representative)
**Key activities**<br>(What are key implementation activities?)	Acquisition of users (try-and-buy-principle)	Session 12 - Interview 28.11.2023: *''Het proberen is misschien nog wel de belangrijkste stap, dus dat het bedrijf aanbiedt dat je het drie maanden op proef kunt krijgen en dat is denk ik ook in het voordeel van het bedrijf om dat te kunnen ondersteunen.''* (age tech company representative)
	Helping users to self-install sensors and app (manual)	Session 11 - Interview 24.11.2023: *''(…) Je kan het ook zo regelen dat iemand dat zelf kan inzetten. Als je die technologie zo makkelijk kan maken dat mensen het zelf kunnen installeren, alsjeblieft doe dat!''* (CEO age tech company)
	Providing user support (helpdesk + Q&A)	Session 4 - Interview 01.11.2023: *''Uitleg geven hoe het werkt via instructies, video's, duidelijke website met veelgestelde vragen, handleidingen.''* (advisor care technology within aged care institution)<br><br>Session 4 - Interview 01.11.2023: *''Ja een paar frequently asked questions dat is fijn of de meeste voorkomende problemen. Maar verder is het meestal: je wil gewoon even iemand spreken zodat je kunt zeggen in jouw eigen woorden wat het probleem is.''* (advisor care technology within aged care institution)

	Certification and medical device registration (if applicable)	Session 5 - Interview 07.11.2023: *''Je moet natuurlijk een CE certificering daarvoor hebben om te laten zien dat het veilig is.''* (health insurer representative)<br><br>Session 7 - Interview 16.11.2023: *''Ja uiteraard, hoe meer certificeringen hoe meer autoriteit. Medical device is een beetje twijfelachtig want je doet niet echt medische uitspraken. Maar inderdaad, dat [de certificering] kan ook wel helpen in de autoriteit.''* (CTO age tech company)
**Key resources**<br>(Which resources are needed to conduct the key activities?	Sensor system and app	N.a. (predefined by scenario)
	Sales website	Session 5 - Interview 07.11.2023: *''Ja maar ik bedoel ook echt even een retailer als in de winkels die je in de straat tegenkomt. Dat kan dus ook bijvoorbeeld een online winkel zijn. Daar zit dus een kostencomponent in en natuurlijk een marge die ze daar krijgen.''* (health insurer representative)
	Trained personnel to execute the key activities	Session 2 - Interview 24.10.2023: *''Wij werken dus aan het lokaal opleiden van facilitaire mensen, die dan dus zorgverleners en mantelzorgers ondersteunen. Want voor een anoniem gezicht van een bedrijf doe je bijna niks…''* (age tech company representative)
	Knowledge of laws regarding privacy (GDPR), ethics (Dutch Care and Coercion Act), and medical devices	Session 3 - Interview 24.10.2023: *''Ja ik denk dat er ook wel echt juridische kennis moet zijn. Privacywetgeving of juridische dingen zijn echt wel van belang dat daar kennis over is. Dus kijken van: welke data mogen wij als bedrijf in dit systeem gaan vastleggen?''* (health insurer representative)<br><br>Session 8 - Interview 20.11.2023*: ''Wij hebben een sensor voor dagritme detectie. Als we zeggen iemand is niet opgestaan dan vallen we onder medical device, als we zeggen er is een ongebruikelijke dagstart dan valt het niet onder een medical device dus je maakt geen conclusie. Je zegt niet iemand is gevallen.''* (age tech retailer representative)
**Cost drivers**<br>(What are the main cost drivers?)	Costs for software and hardware (product development + maintenance)	Session 5 - Interview 07.11.2023: *''Ja je begint natuurlijk met het maken van de oplossing.''* (health insurer representative)
	Internal/ external personnel costs to execute the key activities (user acquisition, user support, certifications)	Session 8 - Interview 20.11.2023: *''Dus als je kijkt naar de kosten vooraf qua telefonisch verkoop of mail of iets, dan zitten er heel veel kosten, want je bent daar veel tijd aan kwijt en de vraag is nog of dat een verkoop wordt. (…) Dus je moet heel erg kijken naar alle verschillende kostenelementen waarbij ook elk telefoontje geld kost zeg maar.''* (age tech retailer representative)<br><br>Session 2 - Interview 24.10.2023: *''De kostenpost waarin veel energie stoppen is helpdesk en klantbeheer. Iemand verhuist misschien wel drie keer, iemand gaat van een groot huis naar een aanleunwoning en gaat toch dan weer een tijdje naar het ziekenhuis.''* (age tech company representative)

		Session 11 - Interview 24.11.2023: *''(…) Als je die technologie zo makkelijk kan maken dat mensen het zelf kunnen installeren, alsjeblieft doe dat! Maar als dat niet zo is en je moet er echt even langs dan zal dat je grootste kostenstructuur zijn.''* (CEO age tech company)
**Revenue streams**<br>(What are suitable funding options?)	Informal caregivers/ PwD: Payment of monthly subscription fee (all-in-price)	Session 1 - Focus group 01.11.2023: *''Zolang de mantelzorger voordelen ziet in het systeem en denkt oh dit gaat mij helpen, hierdoor wordt ik minder ongerust, dan zijn ze wel bereid te gaan betalen.''* (advisor care technology within aged care institution)<br><br>Session 11 - Interview 24.11.2023: *''En dat is misschien dan over je inkomstenstromen, zij kopen de technologie bij ons niet, dus wij hebben een abonnement en zij kunnen de technologie gebruiken en ze betalen voor de service en de dienstverlening waaronder ook een stukje technologie zit maar zij betalen voor het geheel een prijs per maand.''* (CEO age tech company)
**Value proposition for payers**<br>(What are desired outcomes for potential payers?)	Reduction of informal caregiver burden<br>Living at home safely (PwD)	Session 4 - Interview 01.11.2023: *''Ik denk dat het [de technologie] voor de de bewoner zelf rust kan creëren, voor de mantelzorger ook.''* (advisor care technology within aged care institution)

Business model 2: Healthcare market

**BMC element**	**Code**	**Illustrative quote(s) in original language (Dutch)**
**Customer segments**<br>(Who are the potential users and payers?	Potential user:<br>District nurses within home care organizations	N.a. (predefined by scenario)
	Potential user:<br>Informal caregivers & PwD	N.a. (predefined by scenario)
	Potential payer:<br>Health insurers	Session 3 - Interview 24.10.2023: *''In de wijkverpleging, dus dan heb je het over de Zorgverzekeringswet, is er de mogelijkheid om uren te declareren en dan kun je maximaal zes en een half uur per cliënt declareren voor de inzet van de technologie.''* (health insurer representative)
	Potential payer:<br>Long-term care offices	Session 1 - Focus group 01.11.2023: *''Maar ja, qua financiering, je kan ook kijken naar WLZ-VPT, misschien dat daar nog mogelijkheden liggen?''* (advisor care technology within aged care institution)
	Potential payer:<br>Home care organizations	Session 6 - Interview 13.11.2023:<br>*''O: Maar is er dan eigenlijk ook nog een scenario waarin de technologie alleen via het budget van de zorgorganisatie wordt bekostigd? Zoals bijvoorbeeld een EPD, dat wordt natuurlijk ook alleen maar via de zorgorganisatie gefinancierd, omdat het voordeel heeft voor de organisatie.*

		D1: Ja dan moet je dus echt gaan kijken van wat levert het dan op in besparing. (…)’’ (advisor care technology within aged care institution)
**Key partners**<br>(Which key partners are needed?)	Sales partner: Retailer	Session 5 - Interview 07.11.2023: *‘’Ja maar ik bedoel ook echt even een retailer als in de winkels die je in de straat tegenkomt. Dat kan dus ook bijvoorbeeld een online winkel zijn.’’* (health insurer representative)
	Implementation partner:<br>Home care organizations (case managers dementia, policy advisors care innovation)	Session 9 - Interview 22.11.2023:<br>*‘’D: Ik zou de casemanager er altijd een rol in geven.*<br>*O: En dan zou je misschien nog die mensen kunnen bereiken die nog niet in de thuiszorg zitten via een casemanager.*<br>*D: Ja, dat kan. Hoewel ik denk dat het merendeel van de mensen die een casemanager heeft dus ook thuiszorg krijgt.’’*<br>(municipality representative)<br><br>Session 1 – Focus group 01.11.2023:<br>*‘’O: Dus welke partners heeft volgens jullie een bedrijf nodig om deze smart monitoring technologie goed te kunnen implementeren?*<br>*D2: De adviseurs zorginnovatie.’’*<br>(advisor care technology within aged care institution)
	Implementation partner:<br>Electronic client record (ECR) providers	Session 3 - Interview 24.10.2023: *‘’Op het moment dat je het [de technologie] koppelt aan thuiszorginstellingen, kom je in een wat ander perspectief, want dat heb je niet alleen te maken met de thuiszorgorganisatie maar ook met bv. de EPD systemen, dan krijg je direct ook met heel veel randpartijen te maken.’’* (health insurer representative)
	Implementation partner:<br>Care alert system (CAS) providers	Session 1 - Focus group 01.11.2023: *‘’Ja het liefst geïntegreerd. Wij werken hier binnen met [naam zorgoproepsysteem], dat is ons zorgoproepsysteem, alles moet daarop kunnen.’’* (advisor care technology within aged care institution)<br><br>Session 2 - Interview 24.10.2023:<br>*‘’O: In Nederland hebben wij bv de personenalarmering. Zou het misschien een optie zijn om bij zo'n bestaande service aan te haken?*<br>*D: Ja zeker, want dan kan je ook op het moment dat je een alarmering krijgt een beetje kijken wat er aan de hand is.’’*<br>(age tech company representative)
**Channels**<br>(How to reach potential users?)	Case managers dementia	Session 9 - Interview 22.11.2023:<br>*‘’D: Ik zou de casemanager er altijd een rol in geven.*<br>*O: En dan zou je misschien nog die mensen kunnen bereiken die nog niet in de thuiszorg zitten via een casemanager.*

		D: Ja, dat kan. Hoewel ik denk dat het merendeel van de mensen die een casemanager heeft dus ook thuiszorg krijgt.''<br>(municipality representative)
	Policy advisors care innovation within home care organizations	Session 1 – Focus group 01.11.2023:<br>*''O: Dus welke partners heeft volgens jullie een bedrijf nodig om deze smart monitoring technologie goed te kunnen implementeren?*<br>*D2: De beleidsadviseurs zorginnovatie.''*<br>(advisor care technology within aged care institution)
**Customer relationships**<br>(How to maintain relationships with customer segments?)	Online contact (website, email)	Session 4 - Interview 01.11.2023: *''Uitleg geven hoe het werkt via instructies, video's, duidelijke website met veelgestelde vragen, handleidingen.''* (advisor care technology within aged care institution)
	Telephone contact	Session 4 - Interview 01.11.2023: *''Dus op het moment dat jij iemand aan de telefoon hebt, dan kom je waarschijnlijk veel sneller tot een oplossing dan als jij een website hebt waar jij iets intoetst dan kom je waarschijnlijk helemaal niet uit waar je uit moet komen denk ik.''* (advisor care technology within aged care institution)
	Face2face contact (home care organizations)	Session 2 - Interview 24.10.2023: *''Want voor een anoniem gezicht van een bedrijf doe je bijna niks…''* (age tech company representative)
**Key activities**<br>(What are key implementation activities?)	Acquisition of home care organizations	Session 1 - Focus group 01.11.2023: *''Omdat er soms zoveel aanbod is, is het voor ons bijna onoverzichtelijk wat er is aangekomen op de markt. Dus als iemand bereid is naar ons toe te komen en daar uitleg over te geven, dan werkt dat veel fijner dan wanneer je zelf actief gaat zoeken.''* (advisor care technology within aged care institution)
	Creating a business case for home care organizations	Session 2 - Interview 24.10.2023: *''Qua financieringsmodel is het inderdaad complex, en wat ik zie is dat de zorginstellingen zelf niet helder hebben hoe ze zorgtechnologie gefinancierd krijgen, dus één van de dingen die je voor hun moet aanbieden is een heldere business case.''* (age tech company representative)
	Integration with existing care systems (ECRs, CASs)	Session 4 - Interview 01.11.2023:<br>*''O: De thuiszorg werkt natuurlijk al met bepaalde ECD's, bepaalde rapportage systemen. In hoeverre zou je daarbij moeten aansluiten met ons monitoring systeem?*<br>*D: Dat zou wel heel mooi zijn, anders heb je weer twee of drie verschillende apps die je moet openen. En als er inderdaad meldingen in het ECD kunnen komen (…) dat zou wel heel mooi zijn.''*<br>(advisor care technology within aged care institution)
	Training of key users within home care organizations	Session 2 - Interview 24.10.2023: *''Bij een zorginstelling kan jij dus iemand lokaal trainen. (…) Het is geen zorgverlener maar facilitair persoon om zowel implementatie te doen als de verwerving en onderhoud. Dus als het niet werkt, dan komt die persoon ook langs. En dan kan je dus met een bedrijf met relatief weinig mensen de implementatie doen.''* (age tech company representative)

	Providing helpdesk + Q&A	Session 12 - Interview 28.11.2023: *''Sowieso een storingsdienst.''* (age tech company representative)<br><br>Session 4 - Interview 01.11.2023: *''Uitleg geven hoe het werkt via instructies, video's, duidelijke website met veelgestelde vragen, handleidingen.''* (advisor care technology within aged care institution)
	Certification and medical device registration (if applicable)	Session 7 - Interview 16.11.2023: *''Ja uiteraard, hoe meer certificeringen hoe meer autoriteit. Medical device is een beetje twijfelachtig want je doet niet echt medische uitspraken. Maar inderdaad, dat [de certificering] kan ook wel helpen in de autoriteit.''* (CTO age tech company)
**Key resources**<br>(Which resources are needed to conduct the key activities?	Sensor system and app	N.a. (predefined by scenario)
	Sales website	Session 5 - Interview 07.11.2023: *''Ja maar ik bedoel ook echt even een retailer als in de winkels die je in de straat tegenkomt. Dat kan dus ook bijvoorbeeld een online winkel zijn.''* (health insurer representative)
	Trained personnel to execute the key activities	Session 2 - Interview 24.10.2023: *''Bij een zorginstelling kan jij dus iemand lokaal trainen. (…) Het is geen zorgverlener maar facilitair persoon om zowel implementatie te doen als de verwerving en onderhoud.''* (age tech company representative)
	Knowledge of care processes	Session 1 – Focus group 01.11.2023: *''Eigenlijk wil je gewoon een verpleegkundige hebben werken in je bedrijf die de taal spreekt van de zorgorganisatie en die de vertaalslag maakt.''* (advisor care technology within aged care institution)
	Knowledge of laws regarding privacy (GDPR), ethics (Dutch Care and Coercion Act), and medical devices	Session 2 - Interview 24.10.2023: *''Waar je moet oppassen, is natuurlijk de AVG en de wet zorg en dwang. Je mag bijna alles bij iemand monitoren als de persoon daarvoor toestemming geeft, maar bij iemand met afnemende wilsbekwaamheid, is dat heel moeilijk.''* (age tech company representative)<br><br>Session 3 - Interview 24.10.2023: *''Ja ik denk dat er ook wel echt juridische kennis moet zijn. Privacywetgeving of juridische dingen zijn echt wel van belang dat daar kennis over is. Dus kijken van: welke data mogen wij als bedrijf in dit systeem gaan vastleggen?''* (health insurer representative)
**Cost drivers**<br>(What are the main cost drivers?)	Costs for software and hardware (product development + maintenance)	Session 5 - Interview 07.11.2023: *''Ja je begint natuurlijk met het maken van de oplossing.''* (health insurer representative)
	Internal/ external personnel costs to execute the key activities (acquisition, business case creation, technology integration, training	Session 11 - Interview 24.11.2023: *''Ik zou zeggen de kosten zitten in het betrouwbaar maken van de oplossing en het logistieke implementatiegedeelte plus de sales, dat zijn echte 80 procent van de kosten.''* (CEO age tech company)

	activities, user support, certifications)	Session 11 - Interview 24.11.2023: *''Ja voor wat jij doet, ik denk dat de certificeringen een heel klein onderdeel zijn van je kostenstructuur.''* (CEO age tech company)
**Revenue streams**<br>(What are suitable funding options?)	Health insurers:<br>Reimbursement of max. 6.5 extra care hours per PwD per month for home care organizations to finance deployment of the technology	Session 3 - Interview 24.10.2023: *''In de wijkverpleging, dus dan heb je het over de Zorgverzekeringswet, is er de mogelijkheid om uren te declareren en dan kun je maximaal zes en een half uur per cliënt declareren voor de inzet van de technologie.''* (health insurer representative)<br><br>Session 1 - Focus group 01.11.2023: *''Wij bepalen dit kost het eenmalige aanschaf, dit kost het aan bedrag per maand. Wij zijn er zelf ook nog tijd aan kwijt, dus dat verrekenen we dan en dat delen we dan door het aantal cliënten dat we verwachten en dan komt er een tarief uit die wij per maand per cliënt kwijt zijn. Dat rekenen we om in ons uurtarief (…) dus bv. 2 uur per maand per cliënt. Dus dan mogen wij 2 uur per maand per cliënt extra declareren, zodat we daarmee de technologie kunnen betalen. Dat is voor de zorgverzekeringswet.''* (advisor care technology within aged care institution)
	Long-term care offices:<br>Reimbursement (as above) or budget for home care organizations to finance care packages, including deployment of the technology	Session 1 - Focus group 01.11.2023: *''En VPT is dan weer helemaal anders. Dan heb je zo'n rekenmodule waarin je inschrijft wat je allemaal gaat inzetten en daarin zou je technologie ook mee kunnen nemen. Dus in die zak met geld hou je dan reserve over voor de inzet van bijvoorbeeld zo'n product.''* (advisor care technology within aged care institution)
	Home care organizations:<br>Payment from own budget	Session 6 - Interview 13.11.2023:<br>*''O: Maar is er dan eigenlijk ook nog een scenario waarin de technologie alleen via het budget van de zorgorganisatie wordt bekostigd? Zoals bijvoorbeeld een EPD, dat wordt natuurlijk ook alleen maar via de zorgorganisatie gefinancierd, omdat het voordeel heeft voor de organisatie.*<br>*D1: Ja dan moet je dus echt gaan kijken van wat levert het dan op in besparing. (…) Zorgt het ervoor dat je minder vaak hoeft langs te komen in de wijkverpleging of zorgt het ervoor dat je op andere momenten langs kan komen, waardoor je de zorg meer kan spreiden over de dag? Dat zijn dingen waardoor wij kunnen besparen wat dus maakt dat je meer cliënten van zorg kan voorzien.''*<br>(advisor care technology within aged care institution)
**Value proposition for payers**<br>(What are desired outcomes for potential payers?)	Health insurers:<br>Reduction of (nighttime) control visits in home care<br>Prevention of hospital care	Session 1 – Focus group 01.11.2023: *''En bijvoorbeeld in de nacht, kan het ergens zorg vervangen omdat we anders veel vaker langskwamen? En nu is het alleen maar wanneer het echt nodig is. Dan wordt het vergoed door de zorgverzekeraar, daar zijn afspraken voor.''* (advisor care technology within aged care institution)<br><br>Session 4 – Interview 01.11.2023: *''Ik denk dat het voor hun [zorgverzekeraars] op een financieel vlak zeker kosten scheelt doordat er misschien minder zorg ingezet hoeft te worden, minder*

		fracturen, minder insturingen naar het ziekenhuis.'' (advisor care technology within aged care institution)
	Long-term care offices:<br>Reduction of (nighttime) control visits in home care<br>Delayed nursing home admission	Session 9 - Interview 22.11.2023: *''Ik denk dat je iets zou moeten zeggen over hoe lang blijven mensen uit de verpleeghuis setting? Hoeveel langer? Is dat een week? Is dat een maand? Is dat een half jaar? Of is dat een jaar? Want het verpleeghuis kost ongeveer 200 euro per dag. Dat is ongeveer 7000 euro per maand.''* (municipality representative)
	Home care organizations:<br>Improved staff satisfaction<br>Maintaining care provision with fewer staff	Session 4 - Interview 01.11.2023: *''Voor de medewerkers van de thuiszorginstelling moet het een stukje rust brengen dat je weet van als er wat gebeurt, dan krijgen wij die melding wel. Daarnaast hoeft je niet meer iedere nacht te kijken, daarmee stoor je de bewoner ook weer niet.''* (advisor care technology within aged care institution)<br><br>*Session 4 – Interview 01.11.2023: ''Ik denk dat op het moment dat wij dit systeem gebruiken hebben wij minder routes die we hoeven te rijden, dus kunnen we één heel team schrappen.''* (advisor care technology within aged care institution)

Business model 3: Social support market

BMC element	Code	Illustrative quote(s) in original language (Dutch)
**Customer segments**<br>(Who are the potential users and payers?	Potential users:<br>Informal caregivers & PwD	N.a. (predefined by scenario)
	Potential payers:<br>Municipalities	N.a. (predefined by scenario)
**Key partners**<br>(Which key partners are needed?)	Sales partner: Retailer	Session 5 - Interview 07.11.2023: *''Ja maar ik bedoel ook echt even een retailer als in de winkels die je in de straat tegenkomt. Dat kan dus ook bijvoorbeeld een online winkel zijn.''* (health insurer representative)
	Implementation partners:<br>Municipalities and their local collaborators (informal care consultants, assessors support indications)	Session 4 - Interview 01.11.2023: *''Als de gemeente dan dit sensorsysteem op de plank heeft liggen, moet dan niet een consulent van de gemeente inschatten of meneer Jansen daar baat bij heeft?''* (advisor care technology within aged care institution)
**Channels**<br>(How to reach potential users?)	Municipalities and their local collaborators (informal care consultants, assessors support indications)	Session 5 - Interview 07.11.2023*: ''Wat je eigenlijk ook in die gebruiksscenario's ziet is dat een aantal van die segmenten die je nu gedefinieerd hebt, die zou je ook als kanaal kunnen zien. De thuiszorginstelling zou bijvoorbeeld je distributiekanaal kunnen zijn om je propositie bij de*

		mantelzorgers en patiënten te krijgen. Hetzelfde geldt voor gemeenten.'' (health insurer representative)
**Customer relationships** (How to maintain relationships with customer segments?)	Online contact (website, email)	Session 4 - Interview 01.11.2023: *''Uitleg geven hoe het werkt via instructies, video's, duidelijke website met veelgestelde vragen, handleidingen.''* (advisor care technology within aged care institution)
	Telephone contact	Session 4 - Interview 01.11.2023: *''Dus op het moment dat jij iemand aan de telefoon hebt, dan kom je waarschijnlijk veel sneller tot een oplossing dan als jij een website hebt waar jij iets intoetst dan kom je waarschijnlijk helemaal niet uit waar je uit moet komen denk ik.''* (advisor care technology within aged care institution)
	Face2face contact (municipalities)	Session 2 - Interview 24.10.2023: *''Want voor een anoniem gezicht van een bedrijf doe je bijna niks…''* (age tech company representative)
**Key activities** (What are key implementation activities?)	Acquisition of municipalities	Session 9 - Interview 22.11.2023: *''Hier staat de technologie wordt aangeboden door gemeenten die het vervolgens inzetten bij mantelzorgers van thuiswonende mensen met dementie. Zo zou het moeten.''* (municipality representative)
	Training of municipal consultants and indication assessors	Session 4 - Interview 01.11.2023: *''Als de gemeente dan dit sensorsysteem op de plank heeft liggen, moet dan niet een consulent van de gemeente inschatten of meneer Jansen daar baat bij heeft? Hebben zij dan die kennis en kun je hun die kennis bijbrengen?''* (advisor care technology within aged care institution)
	Helping users to self-install sensors and app (manual)	Session 11 - Interview 24.11.2023: *''(…) Je kan het ook zo regelen dat iemand dat zelf kan inzetten. Als je die technologie zo makkelijk kan maken dat mensen het zelf kunnen installeren, alsjeblieft doe dat!''* (CEO age tech company)
	Providing user support (helpdesk + Q&A)	Session 4 - Interview 01.11.2023: *''Uitleg geven hoe het werkt via instructies, video's, duidelijke website met veelgestelde vragen, handleidingen.''* (advisor care technology within aged care institution)<br><br>Session 12 - Interview 28.11.2023: *''Sowieso een storingsdienst.''* (age tech company representative)
	Certification and medical device registration (if applicable)	Session 7 - Interview 16.11.2023: *''Ja uiteraard, hoe meer certificeringen hoe meer autoriteit. Medical device is een beetje twijfelachtig want je doet niet echt medische uitspraken. Maar inderdaad, dat [de certificering] kan ook wel helpen in de autoriteit.''* (CTO age tech company)
**Key resources** (Which resources are needed to conduct the key activities?	Sensor system and app	N.a. (predefined by scenario)
	Sales website	Session 5 - Interview 07.11.2023: *''Ja maar ik bedoel ook echt even een retailer als in de winkels die je in de straat tegenkomt. Dat kan dus ook bijvoorbeeld een online winkel zijn.''* (health insurer representative)

	Trained personnel to execute the key activities	Session 2 - Interview 24.10.2023: *''Wij werken dus aan het lokaal opleiden van facilitaire mensen, die dan dus zorgverleners en mantelzorgers ondersteunen. Want voor een anoniem gezicht van een bedrijf doe je bijna niks…''* (age tech company representative)
	Knowledge of municipal policy	Session 4 - Interview 01.11.2023: *''Als de gemeente dan dit sensorsysteem op de plank heeft liggen, moet dan niet een consulent van de gemeente inschatten of meneer Jansen daar baat bij heeft? (…)''* (advisor care technology within aged care institution)
	Knowledge of laws regarding privacy (GDPR), ethics (Dutch Care and Coercion Act), and medical devices	Session 2 - Interview 24.10.2023: *''Waar je moet oppassen, is natuurlijk de AVG en de wet zorg en dwang. Je mag bijna alles bij iemand monitoren als de persoon daarvoor toestemming geeft, maar bij iemand met afnemende wilsbekwaamheid, is dat heel moeilijk.''* (age tech company representative)<br><br>Session 3 - Interview 24.10.2023: *''Ja ik denk dat er ook wel echt juridische kennis moet zijn. Privacywetgeving of juridische dingen zijn echt wel van belang dat daar kennis over is. Dus kijken van: welke data mogen wij als bedrijf in dit systeem gaan vastleggen?''* (health insurer representative)
**Cost drivers**<br>(What are the main cost drivers?)	Costs for software and hardware (product development + maintenance)	Session 5 - Interview 07.11.2023: *''Ja je begint natuurlijk met het maken van de oplossing.''* (health insurer representative)
	Internal/ external personnel costs to execute the key activities (acquisition, training activities, user support, certifications)	Session 2 - Interview 24.10.2023: *''De kostenpost waarin veel energie stoppen is helpdesk en klantbeheer. Iemand verhuist misschien wel drie keer, iemand gaat van een groot huis naar een aanleunwoning en gaat toch dan weer een tijdje naar het ziekenhuis.''* (age tech company representative)<br><br>Session 11 - Interview 24.11.2023: *''Ik zou zeggen de kosten zitten in het betrouwbaar maken van de oplossing en het logistieke implementatiegedeelte plus de sales, dat zijn echte 80 procent van de kosten.''* (CEO age tech company)
**Revenue streams**<br>(What are suitable funding options?)	Municipalities: Payment for monthly subscription fees of informal caregivers/ PwD	Session 1 – Focus group 01.11.2023: *''De gemeente is wel verantwoordelijk voor de dagstructuur en voor mantelzorg. Dus als je het de technologie gaat inzetten voor mantelzorgontlasting dan zou je echt verwachten dat er vanuit de WMO geld voor is.''* (advisor care technology within aged care institution)<br><br>Session 11 - Interview 24.11.2023: *''(…) En dat is misschien dan over je inkomstenstromen, wij hebben een abonnement en zij kunnen de technologie gebruiken en ze betalen voor de service en de dienstverlening maar zij betalen voor het geheel een prijs per maand.''* (CEO age tech company)

**Value proposition for payers**<br>(What are desired outcomes for potential payers?)	Reduction of informal caregiver burden<br>Cost savings	Session 1 – Focus group 01.11.2023: *''De gemeente is wel verantwoordelijk voor de dagstructuur en voor mantelzorg. Dus als je het de technologie gaat inzetten voor mantelzorgontlasting dan zou je echt verwachten dat er vanuit de WMO geld voor is.''* (advisor care technology within aged care institution)<br><br>Session 9 - Interview 22.11.2023:<br>*''O: En als je als bedrijf een gemeente zeg maar wil overtuigen om in de technologie te investeren, wat voor bewijs zou je dan moeten aanleveren?*<br>*D: Kostenvermindering.''*<br>(municipality representative)
